# Consumers’ Perceptions and Sensory Properties of Beef Patty Analogues

**DOI:** 10.3390/foods9010063

**Published:** 2020-01-07

**Authors:** Jordan Taylor, Isam A. Mohamed Ahmed, Fahad Y. Al-Juhaimi, Alaa El-Din A. Bekhit

**Affiliations:** 1 Department of Food Science, University of Otago, P.O. Box 56 Dunedin, New Zealand; jordan.taylornz@gmail.com; 2 Department of Food Science and Nutrition, College of Food and Agricultural Sciences, King Saud University, Riyadh 11362, Saudi Arabia; iali@KSU.EDU.SA (I.A.M.A.); faljuhaimi@ksu.edu.sa (F.Y.A.-J.)

**Keywords:** acceptability, beef, consumer perceptions, patties, sensory, tempeh

## Abstract

The present study was carried out to gain consumer insights on the use of tempeh (a fermented soy bean product) to improve the healthiness of beef patties and to determine the acceptable level of tempeh (10%, 20%, or 30%) in the patty. The study consisted of conducting two focus groups (*n* = 15), a pilot sensory evaluation, and a full consumer sensory study. The focus groups were asked about their consumption of beef patties, attitudes towards processed meat, attitudes towards negative aspects of red meat consumption, and attitudes towards tempeh consumption, as well as sensory perceptions of the cooked patties and their visual acceptance of raw patties. Focus group discussions suggested that there was a market for the product if consumers were informed of tempeh health benefits. Participants seemed more willing to choose how to balance their diet with an antioxidant source than buy a beef patty with added antioxidants. The focus group participants rated the visual attributes of raw patties from all treatments and it was found that the 20% tempeh and 30% tempeh patties were ranked lower (*p* < 0.05) than the others. Overall, the sensory experiments showed that the inclusion of 10% tempeh was the most acceptable level of addition. There were no significant (*p* > 0.05) differences between the control and 10% tempeh patties for overall acceptability or acceptance of flavor. However, 10% tempeh patties were found to be more tender and juicier than the control (*p* < 0.05). A proper knowledge and awareness of consumers about the benefits of tempeh could allow the development of beef containing tempeh products.

## 1. Introduction

Red meat has been consumed by humans for thousands of years and has played an important part in human evolution. It is a nutrient-rich food which is high in protein; minerals, such as iron, zinc, and selenium; and many vitamins [1]. However, in recent years, there has been a negative consumer reaction to red meat [2]. This has partially been due to its saturated fat content, but also due to the causal link between red meat consumption and the incidence of colorectal cancer. Red meat was found to be correlated with the risk of having colon cancer, whereas fish intake was not associated with the risk and a slight negative association was observed with poultry consumption [3]. The difference between meat types and risk of colon cancer could be due to the high levels of haem in red meat. The catalytic activity of haem iron in promoting oxidative processes may be linked to colon cancer formation. Sesink et al. [4] found that fat alone did not affect the levels of cations found in feces; however haem increased fecal cation concentrations in low, medium, and high fat diets, which demonstrates that haem in the presence of fat impairs the absorption of cations and causes epithelial damage. There was a significant interaction (*p* < 0.001) between haem and fat, which affected the fecal cation concentration [4], supporting a hypothesis for a haem-induced lipid oxidation mechanism as a potential contributor to colon cancer. Much research has been conducted to investigate the addition of non-meat additives or extenders to improve nutritional properties [5,6,7,8,9,10,11,12,13], shelf life [14,15], sensory properties [5,16], and physical parameters [5,16] and make use of by-products of other food industries [5] to improve the quality of meat patties.

Partial substitution of meat by plant products is regarded as an emerging strategy to reduce meat consumption [17,18] and improve the healthiness of meat products. This strategy is successful and accepted by consumers because it is not aiming at eliminating meat from the diet, but targets the implementation of simple adjustment of the products without compromising important attributes desirable in meat [19]. The sensory properties of meat products substituted with plant products are important for the acceptability of products. In particular, taste and texture are highly important characteristics for acceptance [20,21]. The format of the meal [21] and repeat exposure [22] are important for the acceptability of meat substitutes and meat analogues (meat products where a portion of the meat is substituted by another food ingredient, also known as hybrid meat products). Evaluations of meat products substituted with plants [21] or insects [23] have indicated the potential of achieving the same level of acceptability by consumers, but gender differences may exist.

Consumers have become increasingly concerned about fat consumption and have often associated red meat with a high fat content. There are several classes of fat and each contributes differently to the risk of cardiovascular disease [24]. Cardiovascular disease is associated with atherosclerotic plaques which build up on the inside of coronary arteries that provide blood to the heart muscle (myocardium) [25]. These plaques are mainly composed of cholesterol and low density lipoprotein (LDL) particles, which are the main cholesterol carriers. Low density lipoproteins are oxidized and consumed by macrophages [25], which can consequently lead to increased oxidative stress and diseases. The objective of this study was to gain insights on consumers’ perception of the addition of tempeh to beef patties with the aim of producing a healthier beef product, by conducting focus groups, as well as pilot and full consumer sensory trials.

## 2. Materials and Methods

All experiments were approved by the University of Otago Human Ethics committee (09/156).

The experiment design included the following:

(1) Two focus groups (*n* = 15) of subjects that were screened (Section 2.3.1) before joining the focus groups. The focus groups explored the panelists’ attitudes towards the consumption of beef patties, their knowledge of processed meat products, a sensory evaluation of beef patties containing tempeh, and their perception of the new beef analogue after providing information on the health benefits of including tempeh in the product;
(2) pilot sensory analysis study (*n* = 14) to determine the optimum tempeh % (10%, 20%, or 30%) to include in a full-scale consumer sensory study;
(3) Consumer sensory study (*n* = 118) to determine consumers’ perception and acceptability of beef patties containing 10% tempeh compared with a control (100% beef) and a comparably reduced beef patty that contained 10% bread crumb.

### 2.1. Sample Preparation

Soy beans (*Glycine max*) were purchased locally from the Taste nature organic store (Dunedin, New Zealand) and were soaked for 2 days at 4 °C. The beans were cooked for 10 min in a pressure cooker at 100 °C, drained, dried in towels, and treated with white vinegar at a concentration of 2 mL/100 g of beans. A starter culture (*Rhizopus oligosporus*) was then added at a concentration of 1 g culture/kg beans. The mixture was packed in perforated ziplock bags (170 × 180 mm) and incubated in a snaplock container (Klip it, 255 × 120 × 55 mm, 1.75 L, Sistema Plastics, New Zealand) with 1 M potassium nitrate solution to create a humid atmosphere (92% relative humidity) at 31 °C in an incubator (Labserve, Ontherm Scientific Ltd., Hutt City, New Zealand) for 24 h. The produced tempeh was vacuum packed and frozen at −80 °C for further use.

Fresh beef semitendinosus muscles (ST; eye of round, *n* = 6 with total weight of 22 kg) of a normal pH (range 5.55–5.64) were obtained from a local supplier (Alliance Wholesale meats, Dunedin). The meat was separated into lean and fat and then diced. Diced meat and 10% fat were added to a Kenwood blender with a mincing attachment (Alp 5 blade and mincing plate, 4.5 mm diameter die). The patties were prepared (about 4–5 kg for each treatment) as described in Table 1. Five experimental groups (Table 1) were as follows: non treated control sample (control); samples with 10% of the weight replaced with 10% bread crumb (Bread crumb 10%); and samples with part of the weight replaced with tempeh at the level of 10% (Tempeh 10%), 20% (Tempeh 20%), or 30% (Tempeh 30%). Patties were made by shaping 120 g of the mixture with a patty former. Fresh samples were used for color stability trials and other analyses, and the patties were vacuum packed and stored at −80 °C.

### 2.2. Focus Group

Consumer perceptions, including attitudes and sensory perception, are important characteristics of a patty. Focus groups were used for exploratory research to obtain consumers’ insights on the use of plant materials to improve the healthiness of beef patties, determine the level of tempeh inclusion, and serving conditions (preference for at home use vs. take out). This information was used later in designing the consumer sensory analysis and to investigate consumer attitudes towards a novel product such as beef patties containing tempeh.

A focus group is an interview based on a set of predetermined open ended questions which aims to generate discussion amongst participants to gain insights into consumer behavior [26]. It is based on a small number of issues, with the aim of understanding how the behavior of individuals is influenced by their beliefs, attitudes, and feelings [26,27,28].

#### 2.2.1. Participant Recruitment

Flyers were used to recruit participants for two focus groups. They were placed around the University of Otago and Otago Polytechnic campuses, at supermarkets, a public library, and fish and chip shops. Flyers were placed over a period of two weeks and fifteen participants were chosen in total to take part in two focus groups after a brief screening over the phone. Respondents were screened based on three questions, in order to exclude those who would not be eligible for the focus group. The questions were as follows:

1. Are you willing to participate in a recorded discussion on this topic? The recorded data will be handled ethically according to the university policy on private data;
2. Do you have any ethical or religious objections to eating beef?
3. Are you allergic to gluten and/or soy?

Participants who answered yes to question one and no to questions two and three were invited to participate in the focus groups.

The first focus groups attempted to cover the research aspects from diverse age and professional groups, whereas the second focus group sought the opinions of young university students as it was clear that they represent a large fraction of consumers. The characteristics of the focus group participants are shown in Table 2.

#### 2.2.2. Organization of the Focus Group

The focus groups were held on two separate days. The durations of the two focus groups were ninety minutes and eighty minutes for the first and second focus groups, respectively. The focus groups were moderated by the authors. The focus group sessions were held in a sensory lab to encourage interaction and allow for the best audio recording environment. A tape recorder with an external microphone was used to record the answers of participants. Participants read an information sheet and signed a consent form before the sessions. The participants engaged in an ice-breaker discussion with each other and with the moderator for five minutes before the focus group officially started. The focus group session was divided into five parts and guided by the focus group protocol. At the conclusion of the focus group, participants put their name in a basket for a random draw for a prize of a $50 grocery voucher.

#### 2.2.3. Sample Preparation

The five patty treatments were prepared as described in Section 2.2. Patties were then placed in a refrigerator at 4 °C on a tray lined with wax paper; wax paper was put on the surface to prevent drying until they were later cooked on the same day. One patty of each formulation was also placed on polystyrene trays wrapped with glad wrap and stored at 4 °C in a refrigerator until they were later shown to participants for an evaluation of raw patties. The patties were cooked in canola oil on a Kambrook Banquet electric fry pan for two minutes on each side. They were then put into a fan forced oven for ten minutes at 180 °C, which was sufficient to produce an internal temperature >75 °C. The patties were removed, cut into quarters, and wrapped in aluminium foil. They were put into labelled trays and held in an oven at 80 °C until serving within 4–5 min from preparation.

#### 2.2.4. Focus Group Protocol

The focus group protocol consisted of five parts that dealt with the consumption of beef patties, attitudes towards processed meat, attitudes towards negative aspects of red meat consumption, and attitudes towards tempeh consumption, as well as sensory perceptions of the cooked patties and visual acceptance of the raw patties.

Part 1: Attitudes Towards the Consumption of Beef Patties

The first set of questions was about the participants’ normal consumption habits with regards to takeaways, especially beef patties “commonly known as burgers”. Participants were asked about when and where they normally consume beef patties/burgers and what factors influenced their choice of takeaways and/or burgers.

Part 2: Consumer Perception and Knowledge of Processed Meats

At the start of this section, the participants were shown an information sheet on processed meat and the addition of non-meat ingredients to meat products. This section was important for evaluating the consumers’ knowledge of meat extenders and their attitude towards products containing non-meat components as patties may contain up to 30% tempeh. Questions were aimed at exploring whether participants realized how many non-meat ingredients are included in processed meat, understanding why producers do it, and if it seems deceptive or not.

Part 3: Preliminary Sensory Analysis

During this part of the session, the participants analysed the patties for sensory attributes. Quarter sections of patties, temperature tested with a thermometer, were brought from the warming oven in a separate kitchen and were simultaneously served as 3-digit coded samples to participants. The attributes assessed were the intensity of beef odor (slight–strong), intensity of other (non-beef) odor, tenderness (tough–tender), juiciness (very dry–very juicy), chewiness (very chewy–very soft), beef flavor intensity (very slight–very strong), intensity of other (non-beef) flavor (very slight–very strong), acceptance of flavor (dislike extremely–like extremely), and overall acceptance (dislike extremely–like extremely). Attributes were rated on paper ballots with five-point word-anchored scales, with the exception of acceptance of flavor and overall acceptance, which were assessed on seven-point word-anchored scales. This section served as exploratory research for sensory analysis to choose an acceptable level for tempeh inclusion in a patty.

Part 4: Effect of Information of Health Benefits on the Consumer Perception of Novel Beef Patties

This section began by providing participants with an information sheet on published research suggesting the negative aspects of red meat consumption [29] and potential health benefits of tempeh [30,31]. The objective was to see how health information impacts attitudes towards adding a vegetal antioxidant source to the beef patties. Questions during this section were based around previous knowledge of a link between red meat and cancer and if this link led to a change of diet. Participants were also asked if this would increase their likelihood of eating meat with an antioxidant source and if they had tried tempeh. Participants were asked whether they would make an attempt to consume antioxidants with red meat as a separate part of a meal or whether the inclusion of the antioxidant source (such as in the tempeh) in a patty would be a convenient option. They were also asked if they would be willing to purchase a patty containing tempeh.

Part 5: Evaluation of Raw Beef Patties

One patty of each formulation (freshly prepared) was displayed to participants in raw form on a polystyrene tray, as it would normally be presented for retail sale. The objective was to determine the attitudes towards the product in the form it would be sold at retail after the health information had been given. This was decided to be a better method for assessing the purchase intention than consumption of the cooked patties alone, as consumers base meat purchases on visual cues [32,33].

#### 2.2.5. Focus Group Analysis

The focus group discussions were recorded with Audacity software (version 1.2.6) and were later transcribed. Participants and responses were coded and the transcripts were analysed by sorting participant quotes thematically according to the insights they provided into consumer attitudes, in order to write the discussion.

### 2.3. Sensory Analysis

#### 2.3.1. Pilot Sensory Analysis Study

A smaller pilot sensory study (*n* = 14) was carried out before a full-scale sensory study in order to aid in the design of the larger experiment. Participants were students and staff members from the University of Otago.

#### 2.3.2. Consumer Sensory Analysis Study Recruitment

The panelists for sensory analysis (*n* = 118) were recruited by different contact and advertisement methods. The sample size is considered adequate to avoid type I and type II errors that can arise in a consumer sensory test [34]. Panelists were recruited from a database kept by the Food Science Department, University of Otago and from fliers placed around the University campus, Otago Polytechnic campus, and halls of residence; an advertisement at lectures; and by emails circulated by the administrators of university departments. Respondents arranged a time to come in and taste the beef patties and were asked a set of questions to screen out participants unable to participate based on personal beliefs, allergies, or a lack of familiarity with the product. The gender and age categories of panelists are shown in Table 3.

Study Design:

The questionnaire for participants was created in Compusense Five (version 4.8.8, Guelph, Ontario, Canada). The panelists were asked to declare their age and gender categories and for each sample question, they were asked for the attributes of overall acceptability, intensity of beef odor, tenderness, chewiness, juiciness, intensity of flavor, level of non-meat flavor, and acceptance of flavor. After answering these questions for all samples, the consumers were asked about the frequency of beef patty/hamburger and soy product consumption.

Sample Preparation:

Samples were prepared as described in Section 2.1 above for the focus groups. The results obtained from the focus groups and pilot sensory studies guided the treatments chosen for consumer sensory analysis, which included a control, and 10% bread crumb- and 10% tempeh-containing patties. The samples were prepared 1 week before the sensory analysis, frozen at −20 °C, and defrosted in a refrigerator overnight prior to the sensory analysis. The defrosted patties were cooked as described for the focus groups above. After cooking, they were cut into quarters, wrapped in tinfoil, and placed in casserole dishes inside an oven set to 100 °C.

Sensory Analysis:

The analysis was performed in sensory booths in the Sensory Science Research Centre at the Food Science Department of the University of Otago. Participants first read an information sheet and signed a consent form and were then served the samples. The samples were coded with randomized numbers according to the serving order devised by Compusense. The samples were served under ambient light in booths under positive pressure. Participants took one minute breaks and drank water between assessing samples to cleanse their palettes.

### 2.4. Statistical Analysis

The sensory data from the focus groups and pilot sensory study were analysed using a Kruskal–Wallis test (Minitab 16, Minitab, State College, PA, USA). A statistical analysis of sensory attributes obtained in the consumer sensory study was performed as a one way analysis of variance (ANOVA), with treatments as the independent variable, and significant differences were detected at a level of *p* < 0.05, identified by post hoc Tukey tests using Minitab software version 16 (Minitab, State College, PA, USA).

## 3. Results and Discussion

### 3.1. Focus Group

The focus groups were coded for ease of discussion (Supplementary Table S1). The main messages from the focus groups are discussed in the following themes.

#### 3.1.1. Consumption of Takeaways

Most focus group participants consumed takeaways at least once a week and the younger university students generally consumed takeaways more often. The proximity of the takeaway outlet and the price seemed to be the most important factors for an increased consumption of takeaways. Young consumers were reported to be the most frequent takeaway consumers, despite their belief that takeaway food is unhealthy [35]. Younger adult participants have been reported to be more frequent consumers of takeaways than older adults, possibly due to a more positive perception of convenience foods [34,35]. Full time workers consumed takeaways twice as often as non-full time workers [35], although this was not the case in this focus group study.

Two of the second focus group consumers said that they normally buy beef patties/burgers at fast food outlets. Although, in the groups, there was a majority of fast food hamburger consumers, amongst some of the participants in both groups, there was a definite preference for homemade burgers. For example, P2G1 stated that “*they just taste nicer normally, homemade patties and stuff*”, and P9G2 expressed, “*But, I love homemade hamburgers the best*”. One of the reasons mentioned for this is that it was a “*pretty easy meal to prepare*” (P2G1) and “*quite filling as well*” (P1G1). In the second focus group the reason for making homemade burgers were that “*it tastes way better*” (P10G2). There was a noticeable lack of trust in fast food outlets for some of the young female consumers. One reason given for this lack of trust was “*because I know what’s inside*” (P15G2), as the participant studied Human Nutrition papers and had a knowledge of some ingredients used in beef patties and their nutrient contents. Another did not trust that burgers were made from the ingredients that they were claimed to be made from: “*with mince you know it is mince…rather than I don’t know like in chicken burgers, you know is it actually chicken*” (P10G2).

For one participant, it was previous work in the fast food industry which influenced their beliefs: “*You know, how long the meat sits there*” (P9G2). Female consumers preferred to eat burgers from the higher quality takeaway outlets and one stated that they would be “*… willing to pay a bit more for a really good burger*” (P4G1). For the desirable attributes of the hamburgers, younger females placed more emphasis on health, whilst the male consumers did not. One male participant said, “*I don’t think that healthy comes into it when I eat hamburgers personally*” (P1G1). Conversely, two female consumers cited health as one of the desirable attributes for a burger. The meat content was also stated as being important: “*I definitely think that all meat kind of burgers not like probably 30% meat and the rest is other things*” (P15G2).

The consumption of fast food and takeaway food represents a real paradox as consumers mostly find this food to be unhealthy, but its consumption is increasing [36,37,38]. For example, in a previous study, 78% of Americans considered fast food as “not at all good or not too good”, but more than half of Americans were reported as eating fast food at least once a week [36]. The main reasons reported for the frequent use of takeaways and fast food outlets are their convenience, low cost, consistent taste, and easy access, since many outlets are found in localities [36,37,38].

#### 3.1.2. Adding Non-Meat Ingredients and Processed Meat

Overall, the participants were quite skeptical about processed meat and related this to the profiteering of producers: “They are not adding ingredients because they want to make a consumer happy (but) because they see that they can add value to the product” (P6G1). European consumers have also expressed the view that meat processors only work for their benefit, rather than that of the consumer [39]. Some consumers accept this as a way of getting lower priced meat products and are not so concerned. The younger male students in the second focus group are in this category. These products were recognised as a way of selling second-grade meat, which is in agreement with the perception of European consumers [39], although some consumers accept this as part of buying cheaper meat products. Overall, the preference was for non-processed meat forms and was similar to that of the consumers interviewed by de Barcellos et al. [39]. For the second focus group, the word processed had especially negative connotations: “I don’t think I have ever bought the frozen patties because I just think they look so yuck… like it just looks so processed” (P9G2). Additionally, two of the consumers were influenced by having grown up with home-killed meat on a farm.

#### 3.1.3. Sensory Testing of the Beef Patties

One of the panelists was very familiar with tempeh, whilst most were not and used a variety of words to describe the flavor that was foreign to them. It was described as “*vegetablely*” (P2G1), and smelling like “*warm pecans*” (P4G1) or having a “*beaniness*” (P4G1).

#### 3.1.4. The Link Between Red Meat and Colon Cancer

A couple of the panelists had previously heard about a link between red meat and cancer. One was a Human Nutrition student, but was more familiar with the link of heterocyclic amines and processed meats to colorectal cancer. Overall, the panelists were quite skeptical about this link as there were many factors reported as being linked to cancer in the media. In the two separate groups, panelists said, “*everything causes cancer these days*” (P1G1) and “*but they link everything to cancer*” (P10G2). Participants generally did not care or were not willing to change their consumption habits due to this fact and one panelist said, “*it’s more dangerous to dye your hair*” (P8G2). This is in contrast to a separate study, which found that beef consumers were quite health conscious [40], although this study had a more varied age structure. The previous study investigated European consumers and found that a number of consumers had concerns about beef carcinogenicity and its long-term health effects [40].

#### 3.1.5. Consuming Patties with an Antioxidant Source or Balancing Yourself

Participants seemed more willing to balance their diet with an antioxidant source than buy a beef patty with added antioxidants. Some participants perceived this as unnatural and said, “*Normally I would rather, I think, buy something more natural*” (P14G2) and “*I don’t want people chopping and changing my food*” (P11G2). Consumers react negatively towards a perceived ‘interference’ with food products, including the manipulation of beef, which is perceived as ‘unnatural’ and may explain these answers [39,40,41,42]. The ability to sell tempeh patties may be enhanced by the inclusion of a health claim on the packaging as these claims are able to positively influence the consumer perception of a health benefit [43].

However, frequent takeaway consumers are significantly less likely to try to achieve dietary requirements of fruits and vegetables [44], which may make it more difficult to sell tempeh patties through a takeaway outlet. This was stated by the participants: “*to go to eat to McDonalds to have a healthy hamburger, it seems a bit paradoxal*” (P6G1). Participants expressed that a potential consumer would need to be informed of the health benefits in order to be willing to purchase the product: “*If you outlined the ingredients its all like, you need to have that in it maybe, otherwise it would be like why change?*” (P8G2).

#### 3.1.6. Consuming Tempeh

There were contrasting attitudes towards consuming tempeh. Three of the participants had tried it, but those who had not were generally not accepting of the description of tempeh. The description was unappealing for younger consumers not familiar with the product and elicited responses such as “*That doesn’t sound good*” (P14G2) and “*that doesn’t sound appealing*” (P9G2). For an idea of what tempeh was, two participants asked if it was similar to tofu. Food neophobia has a negative effect on the acceptance of functional food products such as beef patties containing tempeh [45]. Food neophobia was not explored in this focus group; however, these consumers may be more neophobic than the public in general. Food neophobia in this group could be due to a lack of exposure to novel and foreign foods.

#### 3.1.7. Evaluation of the Beef Patties

**Figure 1:**
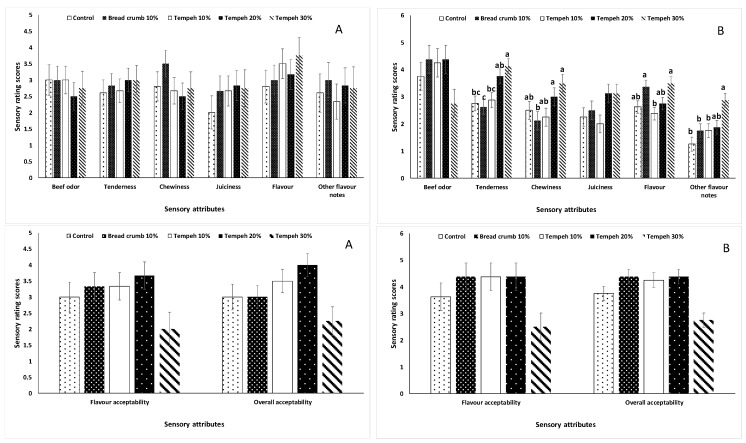
Sensory attribute scores of five beef patties substituted by tempeh at different levels. The patties were evaluated during focus groups (A = focus group 1 and B = focus group 2). ^a–c^ Within each sensory attribute, bars that do not share the same superscripts are significantly different (*p* < 0.05).

**Figure 2:**
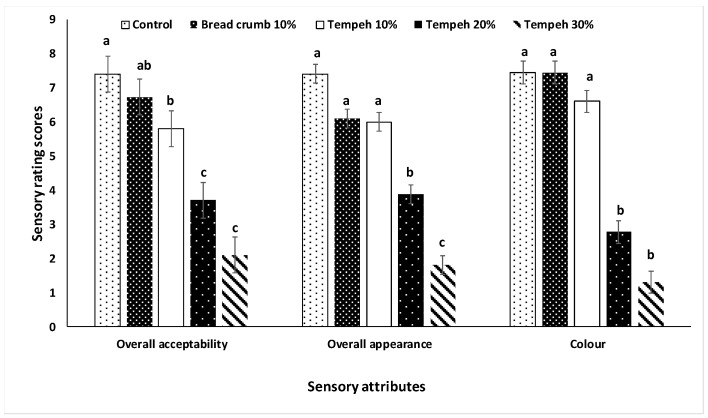
Sensory attribute scores of five raw beef patties substituted by tempeh at different levels. The patties were evaluated by focus groups (*n* = 15). a–c Within each sensory attribute, bars that do not share the same superscripts are significantly different (*p* < 0.05).

The first focus group did not find any differences among all the samples (Figure 1A). For the second focus group, there were significant differences in the tenderness, where the 10% bread crumb was found to be chewy and the least tender, and the 30% tempeh was perceived to be soft and the most tender (Figure 1B). There was no difference (*p* > 0.05) in the intensity of beef flavor among all treatment groups (Figure 1A,B). The participants were asked to choose and rate visual attributes of raw patties from all treatments. The 20% tempeh and 30% tempeh patties were consistently rated lower (*p* < 0.05) than the others (Figure 2). The 10% tempeh patties were not different (*p* > 0.05) from the 10% bread crumb patties and were not significantly different from the control in terms of color or appearance.

There is a relatively large decrease in ratings of visual attributes when increasing tempeh incorporation from 10% to 20% (Figure 2). Ratings of appearance are important as this attribute influences consumer purchase decisions [29,30]. Data from the second focus group suggests that tempeh 10% is the tempeh-containing patty most likely to be purchased. A pilot sensory study was necessary following the focus groups, in order to provide a larger sample size to select an appropriate patty.

### 3.2. Pilot Sensory Study

From the pilot sensory study, it was observed that the 10% tempeh patties had rating scores closer to the control than 20% and 30% tempeh, regardless of significance (Table 4). Most importantly, they were closest in overall acceptability and there were decreased ratings with additional tempeh incorporation (Table 4). This evidence, combined with the focus group quantitative data, which suggested that 10% tempeh patties were the most similar to the control, led to the decision to take the 10% tempeh patty to the full consumer sensory trial. The 10% tempeh patty was more likely to be accepted and less likely to have significant differences in sensory attributes in the larger sample size of the full-size sensory trial than patties containing higher levels of tempeh incorporation.

### 3.3. Consumer Sensory Study

Differences were perceived by participants in terms of flavor and texture sensory attributes, but not for overall hedonic attributes. Mean sensory scores of participants for the overall acceptability and acceptance of flavor did not significantly (*p* > 0.05) differ between the three beef patty treatments (Table 5). The overall acceptability values were lower than in some previous studies with other extenders for tempeh-containing patties [11,46,47]. The substitution of 10% tempeh was more acceptable than the substitution of 3% tomato peel, 2% hazelnut pellicle, or 9% flaxseed flour [5,8,10]; however, it was similar to the substitution of 10% okara powder, an olive oil/corn oil/fish oil blend, 0.5% carrageenan, 6% olive cake, or 7.5% okara, which did not affect the overall acceptability of beef patties [8,11,46,48,49].

The acceptance of tempeh patty flavor was higher than that for patties extended with 4% hazelnut pellicle or 37.5% wet okara, but lower than that for patties extended with carrageen, textured soy protein, tri sodium phosphate, or 10% plum puree [10,11,12,46,47]. Partial substitution of the fat with an olive oil/corn oil/fish oil blend or 3% flaxseed flour similarly did not significantly affect the acceptability of flavor [8,48]. Decreases in flavor acceptability have been observed with the substitution of 3% hazelnut pellicle, 1.5% texturized soy protein, 30% wet okara, and 6% flaxseed flour, and increases have been recorded with the addition of 10% plum puree [8,12,46,47]

Similar to tempeh-containing patties, there were no significant differences in the perception of intensity of beef odor with 15% date fiber [13].

The control was higher (*p* < 0.05) in terms of beef odor than both 10% bread crumb and 10% tempeh (Table 5). Despite the lower beef odor, the overall flavor intensity of 10% bread crumb and 10% tempeh did not differ from the control (Table 5).

The 10% bread crumb treatment was rated the most tender, followed by 10% tempeh and then the control, and all were significantly (*p* < 0.05) different (Table 5). This is in agreement with literature where increased tenderness occurred with the addition of 15% date fiber and 10% carbohydrate-lipid composites [13,50]. The same trend was observed for chewiness. Juiciness was rated the highest for 10% tempeh, while the control was rated significantly (*p* < 0.05) lower and 10% bread crumb did not differ significantly (*p* > 0.05) from either treatment (Table 5). Increases in juiciness with a 10% substitution of carbohydrate-lipid composites or 10% tomato paste have also been reported [50,51]. However, the substitution of up to 30% sorghum flour did not produce any significant difference in juiciness [52]. The use of soy bean products in beef products has been extensively investigated to improve the healthiness of beef products, improve the production economics, or modify the sensory attributes of the products. The use of textured soy protein (TSP) or soy protein concentrate (SPC) at a substitution level of 20% or 30% in beef patties was investigated using a consumer panel and a family consumer panel [53]. The 20% TSP-containing beef patties were rated similar to whole beef patties and the scores for both of these treatments were higher than those found with 30%TSP, and 20% and 30% SPC treatments [53]. Similarly, substitution beef patties with 20% or 30% TSP did not affect consumers’ acceptability, despite the formation of a beany flavor and taste caused by the TSP addition [54]. Additionally, the use of TSP at 25% did not affect the flavor of beef patties [55]. Contrary to these findings, the use of 15% or 30% hydrated TSP in beef patties was found to reduce the beef flavor and overall acceptability of the patties [56], but improved the tenderness of the patties. The same trend was reported for 20% hydrated soy bean [57]. Overall, the various results reported above are likely to be related to differences in the soy bean product, the addition level, and possibly the background of the consumer panel. Unlike many of the studies mentioned above, which used trained sensory assessors [8,10,11,12,13,46,47,50,51,52], this study used a larger untrained consumer sensory trial, which is important for testing the market potential.

## 4. Conclusions

Information from the focus group suggested that consumers are not very concerned with a link between red meat and colon cancer, although several participants had heard of this link. They were skeptical about the media reporting of cancer risks. This gives the impression that there is little potential market for a novel product, such as the tempeh patty; however, the participants were mainly young people, who probably did not have much consideration of a healthy diet. Quantitative data did not show great differences between the patties tested. For appearance, however, 10% tempeh patties were rated closer to the control and bread crumb patties, which suggests that they are more likely to be purchased than other tempeh-containing patties. The 10% tempeh patties had better eating properties. For example, these patties were more tender, juicier, and had more flavor, but they were lower in the intensity of beef odor.

Overall, the 10% tempeh patty was the tempeh-containing patty with the most positive attributes. It was not significantly different than a control patty for overall acceptance and acceptability of flavor and is comparable to a control for visual attributes and more acceptable visually than patties containing more tempeh. The present study did not investigate the impact of tempeh substitution on the volatiles and flavor of the cooked beef patties and future work will address this issue.

## Figures and Tables

**Table 1:** Composition of the five patty treatments.

Treatment	Lean Meat (%)	Fat (%)	Tempeh (%)	Bread Crumb (%)	Salt (%)
Control	89	10	-	-	1
Bread crumb 10%	79	10	-	10	1
Tempeh 10%	79	10	10	-	1
Tempeh 20%	69	10	20	-	1
Tempeh 30%	59	10	30	-	1

**Table 2:** Summary of the characteristics of focus group participants.

Focus Group	Number of Participants	Number of Participants	Number of Participants	Number of Participants	Number of Participants	Number of Participants	Gender Male/Female
	Age ≤18	Age 19–25	Age 25–30	Age 30–40	Age 40–50	Age ≥50	
1	0	1	3	1	1	1	4/3
2	2	6	0	0	0	0	4/4
Total	2	7	3	1	1	1	8/7

**Table 3:** Gender and age group composition of the consumer sensory experiment.

Gender	Age Category					
	18–24	25–30	31–40	41–50	>50	Total
Male	17	12	8	6	5	48
Female	26	8	16	10	10	70
Total	43	20	24	16	15	118

**Table 4:** Mean scores for sensory attributes for five beef patties substituted by tempeh at different levels in the pilot sensory study. The patties were evaluated by a small number of panelists (*n* = 14). ^a–c^ Within each row, means that do not share the same letters are significantly different (*p* < 0.05).

	Control	Bread Crumb 10%	Tempeh 10%	Tempeh 20%	Tempeh 30%	*p* Value
**Overall acceptability**	4.21	4.21	3.93	3.5	2.93	0.155
**Intensity of non-beef odors**	1.71 ^a^	2.57 ^a,b^	2.43 ^a,b^	2.57 ^a,b^	3.00 ^b^	0.045
**Intensity of beef odor**	4.66 ^a^	4.10 ^a,b^	3.94 ^a,b^	3.02 ^b^	2.11 ^c^	0.000
**Tenderness**	2.79 ^a^	4.29 ^c^	2.86 ^a,b^	4.00 ^c^	3.79 ^b,c^	0.000
**Chewiness**	2.57 ^a^	3.86 ^b^	2.36 ^a^	3.57 ^b^	3.57 ^b^	0.000
**Juiciness**	2.57	3.21	2.36	3.14	2.50	0.088
**Flavor intensity**	3.57	3.29	3.43	3.5	3.3 ^6^	0.802
**Non-beef flavor**	1.86 ^a^	2.50 ^a,b^	2.64 ^a,b^	2.64 ^a,b^	3.43 ^b^	0.007
**Acceptance of flavor**	4.29	4.36	3.29	3.71	3.00	0.110

**Table 5:** Mean scores for sensory attributes of five beef patties substituted by tempeh at different levels in the pilot sensory study. The patties were evaluated by a small number of panelists (*n* = 14). ^a–c^ Within each row, means that do not share the same letters are significantly different (*p* < 0.05).

	Control	Bread Crumb 10%	Tempeh 10%	*p* Value
**Overall acceptability**	5.42	5.44	5.38	0.97
**Beef odor**	4.19 ^a^	3.53 ^b^	3.78 ^b^	0.00
**Tenderness**	3.23 ^c^	4.64 ^a^	4.14 ^b^	0.00
**Chewiness**	2.65 ^c^	4.05 ^a^	3.43 ^b^	0.00
**Juiciness**	3.66 ^b^	3.95 ^ab^	4.10 ^a^	0.04
**Flavor intensity**	4.31	4.32	4.25	0.81
**Non-beef flavor**	2.43 ^b^	3.41 ^a^	3.11 ^a^	0.00
**Acceptance of flavor**	5.62	5.60	5.42	0.64

## Supplementary Materials

The following are available online at https://www.mdpi.com/2304-8158/9/1/63/s1, Table S1: Focus group themes for the consumption of takeaways, knowledge of the addition of additives to processed meat products, and relationship between colon cancer and the consumption of processed meat products.
