# Supplementary material for: Consumers’ Perceptions and Sensory Properties of Beef Patty Analogues
Source: Foods. 2020 Jan 7;9(1):63. doi: 10.3390/foods9010063 (PMC7023068; doi:10.3390/foods9010063)
Supplement: Supplementary file 1 [file foods-09-00063-s001.pdf]

Supplementary Table 1. Focus group themes for the consumption of takeaways, knowledge of the addition of additives to processed meat products, and relationship between colon cancer and the consumption of processed meat products.

Question and themes	Focus group A	Focus group B
**Consumption of takeaways**		
How often do you eat takeaways?	P1G1: *I eat takeaways quite a bit. I…this year probably not as much as I usually did, last year I used to pretty much eat them every week. In second year I was twice as bad. Lived in Great King St near McDonalds and everything is handy every single day. Um this year more um more subway more sandwiches but yeah still eat burgers at the flat quite a bit*. I live closer to subway now, that's why. The sub of the day as well… you know its $3.90, a sandwich that fills you up.<br>P2G1: *takeaways probably on average about once or twice a week, not sure yeah living in town there are quite a few options.*<br>P3G1: *A couple of times a week depending on what a takeaway is. So yeah. About once a week from McDonalds*<br>P4G1: *We probably at least once a week, once or twice a week.*<br>P5G1: *Yeah I would say once a week on average.*<br>P6G1: *Once a week*<br>P7G1: *Same*	P8G2: *Three times a week*<br>P9G2: *I am going to go with three times a week, it varies*<br>P15G2: *Probably once a week to twice a* month<br>P10G2: *Sometimes more than three times a week*
How often do you eat burgers, beef burgers?<br>Do you prefer them as takeaway or home-made?	P1G1: *Um well second year it was a lot, I was pretty much eating a Big Mac a day almost (laughter). Yeah now probably we have burgers pretty much once a week and I will probably get McDonalds once a week.*<br>P2G1: *Umm, Oh yeah when we cook them in the flat the home made ones–but I don't eat at McDonalds. Oh they just taste nicer normally, home-made patties and stuff. Quite filling as well.*<br>P2G1: *Yeah, we all like burgers in the flat. So it's a pretty easy meal to prepare and make.*	P10G2: *Bit of a cheese burger fan*<br>8G2: *From a fast food outlet*<br>P10G2: *Yeah fast food outlet*<br>P9G2: *But, I love homemade hamburgers the best. It just because you get to make it yourself and it tastes way better, and good quality stuff*<br>P8G2: *Yeah, good quality yeah*<br>P15G2: *Oh I usually make my own. I don't really like buying burgers outside because I know what's inside*

	P4G1: *Um I don't really eat them, once a week? once every other week?*<br>P5G1: *Yeah, I don't get takeaway burgers very often and probably in the summer we probably have more burgers maybe once a week in the summer. But every few weeks other times.*<br>P4G1: *Now I prefer to eat them at home. We definitely make them, make the patties... so even I mean we use mince in other ways also other than as a burger.*<br>P6G1: *Rarely make at home… , two ,three times per year and I'm with kids forced to go to McDonalds…it would be really difficult for me to eat at home hamburger. But I prefer takeaway from Makimono Food like Japanese food.*<br>P7G1: *I tend to cook them, make burgers more at home than takeaway ones.*<br>P1G1: *Yeah I got pretty addicted to McDonalds aye, I don't know why its just I don't know why, the sauces they use I suppose but yeah*<br>P4G1: *See I think for us if we do buy a takeaway burger the only place I can think of is the Queenstown Fergburger where it's a treat. And you are willing to pay a bit more for a really good burger but for us if we go to McDonalds we wouldn't do that. But we would be willing to pay a bit more for a good burger.*	P9G2: *Yeah, I used to work at McDonalds so I'm just like know, how long the meat sits there and all that*
**Adding non meat ingredients and processed meat**		
What ingredients do you use with the beef patties?	P1G1:*Pineapple usually*<br>P2G1:*Tomato*<br>P1G1:*Tomato,letuce, yeah*	P9G2: *I like getting the right amount of sauce like if its just like if you've got a cheese burger its like oh I don't have enough tomato sauce or like you get to choose a quantity P10G2:Yeah having more than one sauce like tomato sauce and mayonnaise and barbeque sauce or aioli*

		P10G2: *Yeah, aioli…. And when, when you like make your own ones at home and you like can put spices and stuff in the mincemeat rather than just bland*<br>P8G2: *Yeah, carrots, like when you make rissoles and stuff*<br>P9G2: *Carrots are yum*<br>P10G2*: I remember at McDonalds, I think for a limited time they had a burger and it had carrot in the patty….maybe I don't know. And it was square shaped.*
When you include it is, it just for flavour, or is it an effort to make your burger healthy?	P1G1: *Just for flavour I think*<br>P3G1: *Flavour mostly*<br>P2G1: *Yeah it's a nice bit of moisture in your burger, tomato is pretty important. Its crucial*<br>P1G1: *Otherwise its just mince on toast pretty much.*<br>P1G1: *I don't think that healthy comes into it when I eat hamburgers personally*<br>P2G1: *Yeah I put like tomato, lettuce. Like I am making a tasty good burger, bit of crunchiness for texture, yeah I suppose like yeah definitely there is another benefit of health, its important for health and for taste and quality of burger*<br>P3G1: *Texture and nutrition wise*<br>P4G1: *I guess flavour*<br>P5G1: *Yeah I would say flavour, normally the more ingredients the better*<br>P6G1: *Flavour*<br>P7G1: *The nutrition*	P9G2:*Flavour yeah*<br>P11G2:*Bit of texture and flavour P10G2:Ah, I reckon its health reasons. Because you can't taste lettuce, tomatoes disgusting*<br>P13G2: *I think the flavours complement each other well. I like the tomato*
Knowledge of other ingredients being added to processed meat products?	P1G1: *Um not aware of what they are really but I presume that most of the meat I buy would be processed and have other stuff in it. I don't really mind*<br>P2G1: *Yeah, you kind of realise yeah when you are a student you become driven by cost as your main thing. But yeah and*	P9G2: *Yeah, I guess we are quite naïve about it, like you still would eat it and stuff but then someone is like "oh that's really processed".*<br>P8G2: *In a perfect world it would be great not to eat processed stuff but its also a lot cheaper*<br>P9G2: *Yeah. That's just the way it is.*

	you sort of realise that they are just putting stuff in to it but you don't really know what it is or what its effects on you are.<br>P3G1: *Yep I just noticed that um I always thought that soy was added to other things and breads. I don't know.*<br>P4G1: *Yeah I guess you know we accept it, we just buy what we like we don't really care what's in it. yeah*<br>P5G1: *Yeah like I notice on packets of sausages meat content is normally ort of 60-70% so yeah I guess there stuff it is soy or something else*<br>P7G1: *Usually a lot of salt and fat as well*<br>P3G1: *A lot of sodium, salt*	P8G2: *I don't know about you Anna because we both grew up on farms like having home kill and stuff you always made your mincemeat out of whatever was like killed on the farm so like I guess it gave you an aversion to buying stuff that was like so processed*<br>P11G2: *But then it tastes so good sometimes you just can't resist*<br>P8G2:*Yeah, because you get used to it*<br>P15G2:*Yeah, but I don't really consciously eat thinking oh I should eat vegetables because of my worries about cancer*
Do you know why these ingredients have been added?	P4G1: *I mean make it go further and I think that makes it less expensive for consumers, more available and you know flavour, it tastes good.*<br>P5G1:*Yeah that's I would have thought, bring costs down for the product* P6G1*:Youk now of course they are not adding ingredients because they want to make a consumer happy because they see that they can add value to the product*<br>P7G1:*The basic product has not been able to use for anything else so they just found a way to devise these products with some salt and fat and whatever else*	P9G2: *Because its cheaper for them? is it?*<br>P15G2: *It doesn't bother me. I don't really buy sausages or any processed meat I usually buy like proper meat and make whatever I am going to make at home*<br>P9G2: *Yeah, well we just don't take much notices owe wouldn't feel deceived, only if someone pointed it out, you are like oh*
Sensory testing of the beef patties	P1G1: *Smells like meatloaf*<br>P2G1: *Yeah its like vegetablely something*<br>P4G1: *It just smells like warm pecans to me or beaniness*<br>P3G1: *Sort of a woody, smokey smell to me*<br>P2G1: *Yeah, my favourite two were the ones with the most tempeh. I like tempeh, I just found out*<br>P1G1:*Love that tempeh*	P11G2: *prefer the middle two*<br>P13G2*: All taste good except the last one*<br>P10G2: *the last one have strong vege taste*<br>P8G2: *I liked the ones with low temeph*
**The link between red meat and colon cancer**		

Are you aware of the relationship between colon cancer and consumption of processed meat that has been reported in media?	P1G1: *I have heard that before*<br>P2G1: *I think so*<br>P1G1: *Everything causes cancer these days*	P11G2: *You hear like oh I don't know*<br>P13G2*: Some new study/ report*<br>P11G2: *Meat, colorectal cancer, but you hear things about food and cancer all the time*<br>P10G2: *But they link everything to cancer*<br>P15G2: *Mm just a little bit, I've heard about how the meats cooked and the relationship with cancer, especially with barbequing like carcinogenic…and Processed meat and cancer*<br>P12G2:*Yeah,I would be leaning to I'd rather keep the taste and not worry too much about cancer P8G2:Yeah, its more dangerous to dye your hair, but maybe its not cancer, maybe its because we are all fat countries and we don't exercise as well*<br>P14G2*:True*<br>P8G2: *If you are healthy and you have got a balanced lifestyle then you can eat red meat and not worry about it*
Would this information influence your decision on buying red meat or it will not change anything?	P2G1: *Not change anything*<br>P4G1: *We toyed with the idea of being vegetarians but I don't think we can do it. We end up at the supermarket buying more meat.*<br>P7G1: *We go through phases of trying to reduce meat consumption. But it doesn't last very long*<br>P3G1: *I went from vegan to meat and stuff*	P9G2: *Oh I still eat fast food, I don't really care, it all tastes good, you just don't want to think about…*
Consuming patties with antioxidant source or balancing yourself	P7G1: *I would be going for vegetables*<br>P2G1:*Yeah.vegetables*<br>P1G1: *Yeah, I suppose*<br>P2G1: *I think I would factor in my cost and dates…. when making a decision as well*<br>P1G1: *I would probably stick with meat and try to eat more vegetables*	P11G2:*Mmm, nah, I wouldn't personally. Because you can get antioxidants from other things can't you? like broccoli*<br>P13G2:*Nah, probably not*<br>P8G2: *I think it would depend on the taste, if it tastes good, yeah. It depends*<br>P14G2: *Normally I would rather I think something more natural*<br>P13G2:*Yeah I think something natural*

		P11G2: *I don't want people chopping and changing my food*
**Consuming tempeh**		
Do you like the idea of consuming traditional Asian fermented soybean products (Miso, Tofu, or the like)	P7G1: *Not especially*<br>P3G1:*Yep.I do because I really like tofu and miso soup*<br>P2G1: *Yeah it doesn't worry me*<br>P1G1: *Yeah, I suppose I would like to know that it's in the meat but then I probably wouldn't mind if Its actually helping you*<br>P5G1: *I'm ok with it*<br>P4G1: *I guess its like any other thing you eat a processed thing you're not sure what's in it anyhow*<br>P2G1: *You would rather you would know about this and it was like a natural product. Than just stuff to help the people producing it P1G1: Rather than just, yeah to make them make more money.*	P11G2: *I don't like that*<br>P14G2: *That doesn't sound good*<br>P9G2: *That doesn't sound appealing. I think it would put me off*<br>P11G2: *Is that like similar to tofu. What is that like a meat substitute. Put me off*<br>P10G2: *Its really nice fried*<br>P15G2: *Oh yeah, I eat tempeh quite a lot back home. Yeah, its one of my favourite foods*
Would it be acceptable if it is added to beef patty?	P2G1:*Definitely yeah, if the taste was similar probably try to do something that was good for you*<br>P6G1:*Well, you know probably if you find your hamburger containing this ingredient in bakery /deli probably that will be a healthy choice because you go back home, you cook that hamburger and you eat the hamburger you know*<br>P5G1: *I think its not a bad idea for fast food places because I think people who go to the fast food places are not going to bother getting extra vegetables or a salad to go on the side so if it actually makes a difference for health reasons think its quite a good idea*	P14G2:*Depends if I liked it or not, I've never had it before. Yeah, I don't know*<br>P13G2: *Probably. Try it once I suppose*<br>P9G2:*Try it but I would always pick real meat over it*<br>P8G2:*If outlined in the ingredients, you need to have that in it maybe, otherwise it would be like why change*<br>P9G2: *it depends how it complements the rest of the ingredients like you could maybe not notice so much when you are having an actual burger*
